# Health implications of chronic hepatosplenomegaly in Kenyan school-aged children chronically exposed to malarial infections and *Schistosoma mansoni*

**DOI:** 10.1016/j.trstmh.2009.08.006

**Published:** 2010-02

**Authors:** Shona Wilson, Birgitte J. Vennervald, Hilda Kadzo, Edmund Ireri, Clifford Amaganga, Mark Booth, H. Curtis Kariuki, Joseph K. Mwatha, Gachuhi Kimani, John H. Ouma, Eric Muchiri, David W. Dunne

**Affiliations:** a Department of Pathology, University of Cambridge, Tennis Court Road, Cambridge CB2 1QP, UK; b DBL–Centre for Health Research and Development, Faculty of Life Sciences, University of Copenhagen, Thorvaldsensvej 57, 1870 Frederiksberg C, Denmark; c Kenyatta National Hospital, Nairobi, Kenya; d Kenya Medical Research Institute, Nairobi, Kenya; e Kakamega Provincial Hospital, Kakamega, Kenya; f Division of Vector Borne Diseases, Kenyan Ministry of Health, Nairobi, Kenya; g c/o Kenya Medical Research Institute, Nairobi, Kenya

**Keywords:** Malaria, Schistosomiasis, Hepatosplenomegaly, Portal vein, Stunting, Nutritional status

## Abstract

Hepatosplenomegaly among school-aged children in sub-Saharan Africa is highly prevalent. Two of the more common aetiological agents of hepatosplenomegaly, namely chronic exposure to malaria and *Schistosoma mansoni* infection, can result in similar clinical presentation, with the liver and spleen being chronically enlarged and of a firm consistency. Where co-endemic, the two parasites are thought to synergistically exacerbate hepatosplenomegaly. Here, two potential health consequences, i.e. dilation of the portal vein (indicative of increased portal pressure) and stunting of growth, were investigated in a study area where children were chronically exposed to malaria throughout while *S. mansoni* transmission was geographically restricted. Hepatosplenomegaly was associated with increased portal vein diameters, with enlargement of the spleen rather than the liver being more closely associated with dilation. Dilation of the portal vein was exacerbated by *S. mansoni* infection in an intensity-dependent manner. The prevalence of growth stunting was not associated with either relative exposure rates to malarial infection or with *S. mansoni* infection status but was significantly associated with hepatosplenomegaly. Children who presented with hepatosplenomegaly had the lowest height-for-age *Z*-scores. This study shows that hepatosplenomegaly associated with chronic exposure to malaria and schistosomiasis is not a benign symptom amongst school-aged children but has potential long-term health consequences.

## 1 Introduction

In endemic areas, school-aged children bear the greatest burden of infection with *Schistosoma mansoni* and this is associated with subtle morbidities that are distinct from the severe manifestations of hepatic periportal fibrosis, for which the peak prevalence occurs in much older age groups than the peak in *S. mansoni* infection intensities.^1^ Although many of these subtle morbidities and their consequences are difficult to assess or attribute to a single causative agent, they are thought to be major contributors to the disability-adjusted life-years associated with *S. mansoni* infections.^2^ One manifestation of the high infection intensities carried by school-aged children is chronic hepatosplenomegaly in which these organs have a firm to hard consistency when palpated, often being extensively enlarged beyond the costal line,^3^, ^4^ but not associated with ultrasound-detectable hepatic fibrosis.^5^, ^6^

Chronic exposure to malaria can also cause overlapping physical signs of hepatosplenomegaly of a firm consistency in school-aged children,^7^, ^8^ which contributes to what has been described as an epidemic of hepatosplenomegaly in children throughout the tropics.^9^ *Plasmodium*-associated hepatosplenomegaly has been reported as a confounder of *S. mansoni-*associated hepatosplenomegaly,^3^, ^10^ however there is evidence that chronic exposure to malaria and infection with schistosomiasis may interact in childhood hepatosplenomegaly.^11^, ^12^ Studies in Kenya have shown that, as well as being associated with higher *S. mansoni* infection intensities,^6^, ^13^ *S. mansoni-*associated hepatosplenomegaly is more severe in areas where malaria is a greater public health problem, although it is not associated with concurrently detectable parasitaemia.^13^ High levels of IgG_3_ against *P. falciparum* schizont antigen (Pfs-IgG_3_), a marker of relative exposure to malaria and therefore frequency of infection,^14^ are also associated with hepatosplenomegaly.^12^, ^15^, ^16^ Thus, in sub-Saharan Africa hepatosplenomegaly is common amongst school-aged children who are yet to develop immunity to infection with *Plasmodium* spp. or *S. mansoni*, and where exposure to the two infections overlaps geographically hepatosplenomegaly is both more prevalent^13^ and more severe.^12^, ^16^

The adverse effects of this hepatosplenomegaly are not well studied, although it is most prevalent during a critical age in terms of human growth and intellectual development.

Here we present data from a Kenyan school-aged cohort in which hepatosplenomegaly was (a) highly prevalent even in the absence of detectable *S. mansoni* infection, (b) associated with Pfs-IgG_3_ levels and (c) clearly exacerbated in children who were infected with *S. mansoni*.^16^ This allowed us to test whether or not childhood hepatosplenomegaly, in the presence or absence of detectable *S. mansoni* infection, was associated with dilated portal veins and/or stunting of growth. A school feeding programme, introduced in 1999, ensured that the study was not confounded by current poor protein and micronutrient intake.

## 2 Materials and methods

### 2.1 Study area and population

The study area in Makueni District, Kenya, in which *S. mansoni* transmission was restricted to the east owing to habitat availability for the *Biomphalaria* snail intermediate host but where *P. falciparum* was transmitted throughout, as well as the selection of school-aged children (4–17 years) from two primary schools who participated in the study are described in detail elsewhere.^16^ Informed consent was obtained from parents or guardians. Five stool samples were collected from participating children and two 50 mg Kato–Katz slides^17^ were prepared from each stool sample. A child was considered free of *S. mansoni* infection if all 10 slides were negative for *S. mansoni* eggs. All *S. mansoni* infections were treated with a single dose of praziquantel 40 mg/kg body weight. Pfs-IgG_3_ levels were measured by ELISA as described previously.^14^ Malaria transmission is considered to be mesoendemic due to highly seasonal rains, with prevalence amongst schoolchildren of microscopy-detectable *P. falciparum* infections being recorded as 15.3% at the end of the long dry season, rising to 51.8% during the high transmission season.^14^ At the time of the study, the prevalence of microscopy-detectable *P. falciparum* infections was 21.0% at one primary school and 19.6% at the other and was not associated with hepatosplenomegaly.^16^ The field work was carried out in May–June 2002.

### 2.2 Clinical examination

Children were examined clinically for palpable, enlarged livers and spleens of a firm consistency and were classed into groupings of (a) no organomegaly, (b) splenomegaly only, (c) hepatomegaly only and (d) hepatosplenomegaly, as described previously.^16^ This variable is referred to as ‘clinical group’. Clinical measurements of the left liver lobe and spleen were also classed as an ordinal variable for extent of organomegaly. The first category was no enlargement of the organ. The left liver lobe was considered moderately enlarged if palpable 3–5 cm and substantially enlarged if palpable >5 cm below the costal margin in the liver mid-sternal line. The spleen was considered moderately enlarged if palpable 3–4 cm below the costal margin in either the mid-clavicular or mid-axillary line or substantially enlarged if palpable >4 cm below the costal margin in either line.

### 2.3 Ultrasound examination

A randomised cohort of 272 children aged 4–17 years was selected from two primary schools in the area, Yumbuni Primary to the west and Matangini Primary to the east, to participate in ultrasound examinations. The children were examined using an Aloka SSD-500 portable ultrasound machine with a 3.5 MHz curvilinear (60%) probe (Imai, Tokyo, Japan). Ultrasound examinations were conducted according to the Niamey protocol^18^ and included measurements of the portal vein diameter (PVD), taken in the right oblique view, at the point of entrance into the porta hepatis at the ventral lower end of the caudate lobe; the measurement taken was the distance between the inner sides of the walls. Ultrasound measurements of PVD required height standardisation prior to analysis. As no data are available from a suitable reference population, standardisation was carried out internally by linear regression. The appearance of the liver parenchyma was assessed in the substernal transverse and subcostal transhepatic views, and liver scores were used to assign fibrosis scores to the appearance observed. Score ‘C’ and above on a scale of A to F are considered indicative of periportal fibrosis. The presence of portosystemic collaterals and ascites was recorded if found.

### 2.4 Anthropometric examination

Heights were measured to the nearest eighth of an inch and converted into centimetres. Weight was measured to the nearest half kilogram. Trained local demographers interviewed adult family members to determine the year of birth of participating schoolchildren. The mid-point of the year was assigned as the month of birth for calculation of *Z*-scores. To compare anthropometric measurements with international standards, height-for-age *Z*-scores (HAZ) and body mass index (BMI)-for-age *Z*-scores (BMIZ) were calculated using the CDC 2000 standards and NutStat software (http://www.cdc.gov/epiinfo/).

### 2.5 Statistical analysis

Complete parasitological, serological, clinical and anthropometric data were available for 353 children. Wasting and stunting were assigned using the respective WHO-defined cut-offs of BMIZ < –2 and HAZ <–2. Proportions were compared using Pearson's χ^2^ analysis and continuous variables were compared by Student's *t*-test and ANOVA with Hochberg GT2 post-hoc analysis. Pearson's correlation coefficients were calculated, except in relation to *S. mansoni* infection intensities when non-parametric Spearman's rank correlation coefficients were calculated. Logistic regression models were constructed using presence or absence of stunting as the dependent variable. Analysis of co-variance (ANCOVA) models were constructed using HAZ as the continuous dependent variable.

## 3 Results

### 3.1 Ultrasound image patterns and portal vein diameter measurements

No *S. mansoni* eggs were detectable in the stool samples provided by 158 of the children examined by ultrasound. Amongst the 114 children who did have detectable *S. mansoni* eggs, the infection intensity ranged from 2 eggs per gram of faeces (EPG) to 713 EPG, with a median infection intensity of 35.42 EPG. No hepatic periportal fibrosis was detected during the ultrasound examinations in either group, with 87.3% of children without detectable *S. mansoni* infections and 71.9% of children with detectable *S. mansoni* infections having pattern A scans; all other children had pattern B scans. Neither ascites nor collateral veins were detected during either the clinical or the ultrasound examinations.

**Figure 1:**
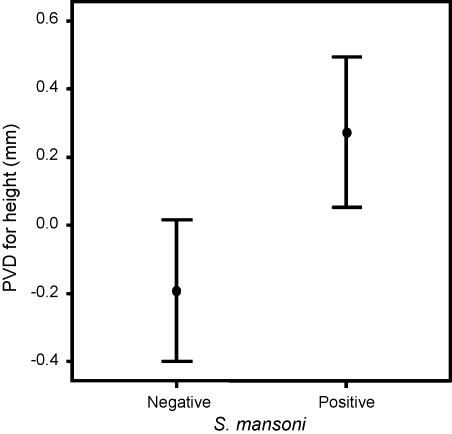
Portal vein diameters (PVD) by *Schistosoma mansoni* infection status. Shown are the mean ± 2 standard errors of the height-adjusted PVDs for children with and without *S. mansoni* infection.

There was no significant difference in the prevalence of organomegaly between children with and without detectable *S. mansoni* eggs (χ^2^ = 1.492, *P* = 0.684). However, height-adjusted PVD measurements were found to be greater in children who had detectable *S. mansoni* eggs than in those who did not (*t* = –3.165, *P* = 0.002) (Figure 1). Height-adjusted PVDs of children with detectable *S. mansoni* eggs were positively correlated with *S. mansoni* infection intensities (ρ = 0.261, *P* = 0.005). Pfs-IgG_3_ levels were significantly correlated with height-adjusted PVD measurements both in *S. mansoni* egg-negative and egg-positive children (*r* = 0.214, *P* = 0.008; and *r* = 0.211, *P* = 0.040, respectively).

**Figure 2:**
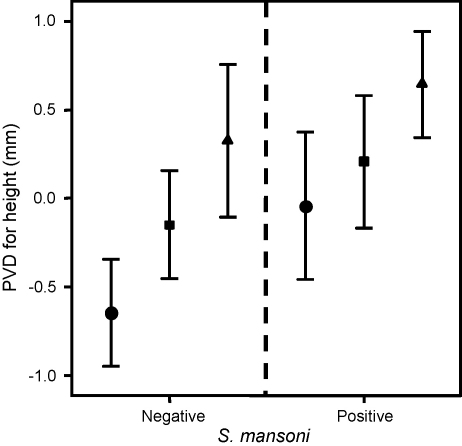
Portal vein diameters (PVD) by *Schistosoma mansoni* infection status and extent of splenomegaly. Shown are the mean ± 2 standard errors of the height-adjusted PVDs for children with differing extents of splenomegaly. Results are shown separately for children with and without *S. mansoni* infection. ●, Spleen palpable 0–2 cm below the costal line; ■, spleen palpable 3–4 cm below the costal line; and ▴, spleen palpable >4 cm below the costal line.

Children without detectable *S. mansoni* eggs but with hepatosplenomegaly had significantly greater height-adjusted PVDs than *S. mansoni* egg-negative children with enlargement of the liver only (*F* = 4.622, *P* = 0.004; post-hoc, *P* = 0.010). Post-hoc analysis indicated that children with hepatosplenomegaly also had greater height-adjusted PVDs than children with enlargement of neither organ, but this failed to reach significance (*P* = 0.067). No significant relationship was seen between height-adjusted PVD measurements and clinical groupings of organomegaly (*F* = 2.015, *P* = 0.117) in *S. mansoni* egg-positive children. Amongst *S. mansoni* egg-negative and egg-positive children there was no significant difference in height-adjusted PVDs between children who had no, moderate or substantial enlargement of the left liver lobe (*F* = 1.566, *P* = 0.212; and *F* = 2.84, *P* = 0.063, respectively). However, height-adjusted PVDs did differ significantly between children with no, moderate and substantial enlargement of the spleen. Post-hoc analysis indicated that height-adjusted PVDs were greater for children with substantially enlarged spleens compared with children with no enlargement of the spleen (*S. mansoni* egg-negative, *F* = 7.852, *P* = 0.001; *S. mansoni* egg-positive, *F* = 3.797, *P* = 0.025) (Figure 2).

### 3.2 Anthropometry

Of the children participating in the study, 56.4% (*n* = 199) were stunted according to international standards. Poor BMI was not as prevalent as stunting, with 25.2% (*n* = 89) of the children examined found to be wasted according to international standards. None of the parasitological or clinical variables were significantly associated with BMIZ (data not shown). A higher proportion of children with *S. mansoni* infections were stunted compared with those who did not have detectable *S. mansoni* infections [61.7% (79/128) vs. 53.3% (120/225)], but this did not reach significance (χ^2^ = 2.333, *P* = 0.127). *Schistosoma mansoni* egg-negative children who were stunted had higher Pfs-IgG_3_ levels (*t* = –3.647, *P* < 0.001) than those who were not stunted. A similar trend was observed for children who were *S. mansoni* egg-positive, but this failed to reach significance (*t* = –1.368, *P* = 0.174).

**Figure 3:**
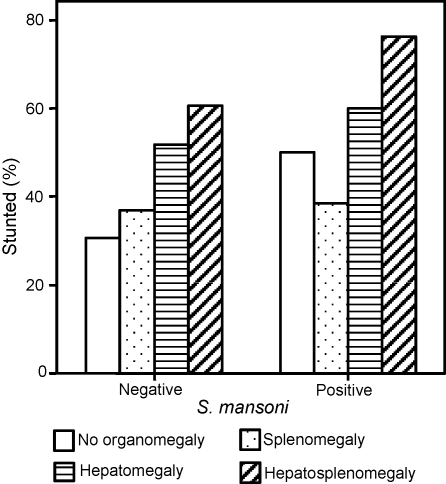
Proportion of children who were stunted, by *Schistosoma mansoni* infection status and clinical groupings of organomegaly. Shown are the proportion of children who were stunted according to international classification (height-for-age *Z*-score <2) in each of the clinical groups. Results are shown separately for children with and without *S. mansoni* infection.

Both for *S. mansoni* egg-negative and egg-positive children there was a significant association between being stunted and clinical groupings of organomegaly (χ^2^ = 9.160, *P* = 0.027; and χ^2^ = 10.401, *P* = 0.015, respectively). Those presenting with hepatosplenomegaly had the highest rates of stunting (Figure 3). A logistic regression model was constructed to control for age and sex (Table 1) and it was found that boys were more likely to be stunted than girls and that the prevalence of stunting increased with age. After controlling for age and sex as well as relative exposure to malaria and schistosomiasis infection status, clinical group remained a significant predictor of stunting. Parameter estimates failed to indicate in which clinical groups the prevalence of stunting was significantly increased. However, the prevalence of stunting amongst children with hepatosplenomegaly compared with children without organomegaly was of borderline significance.

**Table 1:** Logistic regression analysis of stunting of growth

Variable	*P*-value	Odds ratio (95% CI)
Sex	<0.001	2.734 (1.691–4.421)
Age	<0.001	1.234 (1.148–1.373)
*Schistosoma mansoni*	0.678	
Pfs-IgG_3_	0.113	
Group:	0.001	
Splenomegaly only	0.15	0.472 (0.169–1.314)
Hepatomegaly only	0.109	2.011 (0.859–4.709)
Hepatosplenomegaly	0.061	2.264 (0.963–5.322)

Pfs: *Plasmodium falciparum* schizont antigen. Model fit: χ^2^ = 76.612, *P* < 0.001; Nagelkerke pseudo-*R*^2^ = 0.262.

To examine the association between clinical group and stunting further, ANCOVA was carried out using HAZ as the dependent variable. The model was controlled for age (*F* = 83.077, *P* < 0.001) and sex (*F* = 7.333, *P* = 0.007), and clinical group was added as a fixed factor (*F* = 5.341, *P* = 0.001). Pairwise comparisons between groups, adjusted for multiple comparisons, are shown in Table 2. Children with hepatomegaly only and those with hepatosplenomegaly had significantly lower HAZ scores than children with splenomegaly only. There was a borderline significance in lower HAZ for children with hepatosplenomegaly compared with children with no organomegaly.

**Table 2:** Pairwise comparisons of height-for-age *Z*-scores between children in different clinical groups^a^

Clinical group		Mean difference (95% CI)	*P*-value
No organomegaly vs.	splenomegaly	−0.161 (−0.873, 0.551)	0.992
	hepatomegaly	0.480 (−0.131, 1.091)	0.209
	hepatosplenomegaly	0.558 (−0.001, 1.124)	0.056
Splenomegaly vs.	hepatomegaly	0.641 (0.036, 1.246)	0.032
	hepatosplenomegaly	0.791 (0.160, 1.278)	0.004
Hepatomegaly vs.	hepatosplenomegaly	0.078 (−0.327, 0.482)	0.997

a Pairwise comparisons between clinical groupings are shown after controlling for age and sex by analysis of co-variance and adjusting for multiple comparisons.

## 4 Discussion

Although there are many known aetiological agents of hepatosplenomegaly in school-aged children, two of the most common are *S. mansoni* infection and chronic exposure to malaria. Two potential consequences of hepatosplenomegaly in school-aged children, namely dilation of the hepatic vascular system (which could be indicative of increases in portal pressure) and stunting of growth, were investigated in this paper.

In a previous study of children presenting with hepatosplenomegaly without ultrasound-detectable fibrosis, 28% were found to be classifiable as having portal hypertension under the current Niamey protocol.^6^ However, the hepatosplenomegaly that these children presented with was also associated with Pfs-IgG_3_ levels,^12^ a marker of chronic exposure to malaria.^14^ Early histological studies on biopsy specimens from children with malaria-associated hepatomegaly indicate that this may also be accompanied by dilation of the liver's vascular system.^9^ Ultrasound PVD measurements, taken to assess *S. mansoni-*associated pathology, could therefore be confounded by chronic exposure to *Plasmodium* infections. In the present study, amongst the children who did not have detectable *S. mansoni* infections, the height-adjusted PVDs were greatest in those children who presented with hepatosplenomegaly, indicating that the presence of organomegaly in the absence of detectable *S. mansoni* infection was associated with dilation of this vein. However, analysis of the height-adjusted PVD measurements of children with different extents of hepatomegaly or splenomegaly suggests that enlargement of the spleen, rather than enlargement of the liver, was the major contributor to dilation of the portal vein. The height-adjusted PVDs were also significantly related to Pfs-IgG_3_ levels. Whether dilation of the portal vein was due to *P. falciparum*-associated enlargement of the liver or was due to downstream consequences of increased demand for blood by the enlarged spleen cannot be determined from the present study. However, this study does indicate that in areas where there is mesoendemic seasonal transmission of malaria, resulting in a high prevalence of hepatosplenomegaly in school-age children, dilation of the portal vein can occur.

Detectable *S. mansoni* infection was found to exacerbate dilation of the portal vein. The exacerbation of portal vein dilation was *S. mansoni* infection intensity dependent, concurring with a previously reported study involving a morbidity-only cohort of Akamba children, selected on the basis of having hepatomegaly,^6^ as well as a cross-sectional study of Egyptian children.^19^ During earlier surveys, some *S. mansoni-*infected children from Machakos, now Makueni District, Kenya, were found to have the severe complication of portal hypertension and had to be hospitalised,^13^ and upon admission a subset were found to have oesophageal varices in the absence of periportal fibrosis. However, the ultrasound examination was part of the overall clinical examination and did not follow the current standardised protocols (E. Ireri, unpublished observations). The study by Vennervald et al.^6^ and the present study have both applied the standardised Niamey protocol for ultrasound examination^18^ and the implication from these studies is that even when ultrasound-detectable periportal fibrosis is absent, increases in portal pressure related to *S. mansoni* intensity may occur. Further assessment using techniques such as Doppler ultrasound, which would allow determination of blood flow rate and direction, should be implemented to confirm whether the exacerbation of portal vein dilation by *S. mansoni* infection is due to increases in portal pressure.

Unlike dilation of the portal vein, the prevalence of stunting was not found to be significantly increased by the presence of *S. mansoni* infection, and the significantly higher levels of Pfs-IgG_3_, a marker of age and geographical exposure to *Plasmodium* infections, amongst children who were stunted was no longer apparent after controlling for age and sex. However, children with hepatosplenomegaly did have the highest prevalence of stunting in comparison with children who were assigned to the other clinical groups, and HAZ scores were significantly lower in children with hepatosplenomegaly and hepatomegaly after controlling for sex and age. Older children were more likely to be stunted than younger children. Although the use of cross-sectional data can be misinterpreted due to environmental factors during early life causing long-lasting effects on HAZ,^20^ poor growth increments have been reported during the later stages of childhood and adolescence,^21^ indicating that deficits in growth continue to occur amongst this age group. The increase in degree of stunting that occurs with age amongst children with hepatosplenomegaly is in concurrence with a previous study in which internally standardised height-for-age decreased with age amongst Akamba children with hepatomegaly.^22^ Boys were found to be more stunted than girls. In concurrence with this, a meta-analysis of studies, albeit of younger children, has shown that in sub-Saharan Africa boys have higher rates of stunting than girls.^23^

Between-household variation in social and economic factors, which will impact both on nutritional intake in the children's diet and their exposure to infectious diseases, were not controlled for and are confounders that cannot be ruled out. A Brazilian study has shown that *S. mansoni-*infected children presenting with palpable spleens still have poorer height-for-age after controlling for socioeconomic factors.^24^ However, direct extrapolation from this Brazilian study in a non-malarial region is not possible, as presentation with *S. mansoni*-associated splenomegaly in areas of non- or low-endemicity for *Plasmodium* spp. is likely to be indicative of an alternative underlying mechanism of splenomegaly to that present in the current study where malaria is mesoendemic. Current levels of wasting were at a relatively low level in comparison with stunting, which may be attributable in part to the introduction of the school feeding programme that ensures good levels of micronutrient and protein intake.

It has been proposed that chronic inflammation, and in particular production of TNFα and IL-6, may have a direct impact on the linear growth of children by affecting bone remodelling.^25^ An inflammatory response localised within the liver could also inhibit linear growth, as the inflammatory cytokine TNFα can reduce the production of insulin growth factor-1 (IGF-1), a hormone involved in linear growth, by the liver.^26^ Thus, this is a potential mechanism for the strong association between hepatomegaly and stunted growth that was observed, particularly as hepatosplenomegaly within Akamba children has been found to be associated with a pro-inflammatory Th1 response^27^ and low levels of regulatory cytokines.^28^ Low levels of IGF-1 have been shown to be associated with the presence of hepatosplenic schistosomiasis, indicative of periportal fibrosis, in Brazil.^29^ However, it remains to be determined whether or not there is an association between IGF-1 levels and childhood hepatosplenomegaly in the absence of periportal fibrosis.

In conclusion, this study has shown that children with hepatosplenomegaly had both greater dilation of their portal veins, which could be indicative of increases in portal pressure, and higher rates of stunting of growth. Both consequences were apparent in *S. mansoni* egg-negative children with hepatosplenomegaly, highlighting the largely ignored public health implications of long-term exposure to *Plasmodium* infections, and therefore recurrent infections with *Plasmodium*, suffered by school-aged children. Children who were infected with *S. mansoni* and chronically exposed to malarial infections, a group who have exacerbation of hepatosplenomegaly, had greater dilation of the portal vein, which was dependent both on *S. mansoni* infection intensity and the extent of spleen enlargement. This indicates that where exposure to both parasites is occurring, the health consequences as well as the underlying hepatosplenomegaly are exacerbated. Hence, the study shows that persistent firm-to-hard hepatosplenomegaly associated with chronic exposure to malaria and *S. mansoni* infection is not a benign symptom amongst school-aged children but has potential long-term health consequences.

## Funding

Wellcome Trust, London, UK (grant nos. 065478/Z/01/A and 074961/Z/04/Z).

## Conflicts of interest

None declared.

## Ethical approval

The Kenya Medical Research Institute Ethical Review Committee approved the study.

## Authors’ contributions

SW undertook the serology and analysis and drafted the manuscript; BJV conducted the clinical examinations, participated in the design of the study and critically revised the manuscript; HZ and EI conducted the ultrasound examinations; CA conducted the clinical examinations; MB participated in the design of the study and in fieldwork; JKM, GK, HCK, JHO and EM participated in the planning and execution of field activities; DWD participated in the design of the study and critically revised the manuscript. All authors read and approved the final manuscript. SW is guarantor of the paper.
